# Electro-Homœopathy

**Published:** 1893-07

**Authors:** William Steinrauf

**Affiliations:** St. Charles, Mo.


### ELECTRO-HOMŒOPATHY.

William Steinrauf, M. D., St. Charles, Mo.

The sale of the genuine and pseudo Mahei remedies has been prohibited by law in all Austria as a species of humbug and quackery, and advertisements of the same remedies are now forbidden in several of the German principalities, according to the last number of a Leipzig medical journal. That is right. The law holds that they are secret remedies, and as such must be treated.

In this country they are fast losing the little hold they ever had, and our pharmacies are ceasing to advertise them. They only harm our cause. They are no "improvement on Homœopathy." Away with them.

